# Women’s Access to Kidney Transplantation in France: A Mixed Methods Research Protocol

**DOI:** 10.3390/ijerph192013524

**Published:** 2022-10-19

**Authors:** Latame Adoli, Maxime Raffray, Valérie Châtelet, Cécile Vigneau, Thierry Lobbedez, Fei Gao, Florian Bayer, Arnaud Campéon, Elsa Vabret, Laëtitia Laude, Jean-Philippe Jais, Eric Daugas, Cécile Couchoud, Sahar Bayat

**Affiliations:** 1 Université de Rennes, EHESP, CNRS, INSERM, Arènes–UMR 6051, RSMS–U1309, 35000 Rennes, France; 2 U1086 INSERM, Anticipe, Centre de Lutte Contre le Cancer François Baclesse, Centre Universitaire des Maladies Rénales, 14000 Caen, France; 3 IRSET (Institut de Recherche en Santé, Environnement et Travail), Université de Rennes, Chu Rennes, INSERM, EHESP, UMR_s 1085, 35000 Rennes, France; 4 Renal Epidemiology and Information Network (Rein) Registry, Biomedecine Agency, Saint-Denis-la-Plaine, 93212 Paris, France; 5 Arènes–UMR 6051, ISSAV, EHESP, CNRS, 35000 Rennes, France; 6 Service de Néphrologie, Chu Rennes, 35000 Rennes, France; 7 Unité de Biostatistique, Hôpital Necker-Enfants Malades, AP-HP, Institut Imagine, Université Paris-Cité, 75015 Paris, France; 8 INSERM U1149, Université Paris Cité, Assistance Publique-Hôpitaux de Paris, Service de Néphrologie, Hôpital Bichat, 75018 Paris, France

**Keywords:** chronic kidney disease, access to care, mixed methods, gender, inequalities, spatial analysis

## Abstract

Kidney transplantation is the best renal replacement therapy (medically and economically) for eligible patients with end-stage kidney disease. Studies in some French regions and in other countries suggest a lower access to the kidney transplant waiting listing and also to kidney transplantation, once waitlisted, for women. Using a mixed methods approach, this study aims to precisely understand these potential sex disparities and their causes. The quantitative study will explore the geographic disparities, compare the determinants of access to the waiting list and to kidney transplantation, and compare the reasons and duration of inactive status on the waiting list in women and men at different scales (national, regional, departmental, and census-block). The qualitative study will allow describing and comparing women’s and men’s views about their disease and transplantation, as well as nephrologists’ practices relative to the French national guidelines on waiting list registration. This type of study is important in the current societal context in which the reduction of sex/gender-based inequalities is a major social expectation.

## 1. Background

The Kidney Disease Improving Global Outcomes (KDIGO) guidelines define kidney disease as the presence of functional and/or structural abnormalities of the kidney with implications for health [1]. Chronic kidney disease (CKD) is defined by the detection of kidney damage markers or decreased glomerular filtration rate (GFR) that persists for >3 months, and is classified according to the cause, GFR, and albuminuria [1,2]. End-stage kidney disease (ESKD) (i.e., estimated GFR < 15 mL/min/1.73 m²) can be treated by renal replacement therapies (RRT) (hemodialysis, peritoneal dialysis, or kidney transplantation) or conservative care [3,4]. Compared with dialysis or conservative care, kidney transplantation is associated with a longer lifespan, better quality of life, and lower costs for the eligible patients who accept to undergo this procedure [3,5,6,7,8,9,10,11,12,13,14,15,16,17,18]. In France, the first step towards kidney transplantation (from a living or deceased donor) is the patient’s registration on the national kidney transplant waiting list by a nephrologist in accordance with the patient’s choices and after confirming the absence of contraindications [19]. Once waitlisted, access to transplantation depends on a French national organ allocation score that was developed by the National Agence de la Biomedicine using several parameters, such as the recipient–donor age, HLA and blood type matching, and waiting time on the list [19,20]. Access to kidney transplantation also depends on organ availability, on transplantation activity organization within centers [21], and on the patient’s inactive status periods on the waiting list. Indeed, all waitlisted patients who are currently unavailable or unsuitable for transplantation are identified by a temporary inactive status and cannot be transplanted [22,23].

Previous studies have identified several medical and non-medical factors that limit access to kidney transplantation at each step of the process. Patients’ characteristics, such as comorbidities [24,25,26,27], diabetic nephropathy [21,23,24,25,28], and an older age [21,23,24,25,26,27,29], are factors that limit waitlisting and access to kidney transplantation once waitlisted [21,24,25,29]. Moreover, several studies have shown that when compared with men and after adjustment for age and comorbidities, access to the transplant waiting list and to kidney transplantation is lower for women [24,25,27,28,29,30].

Sex/gender disparities in the access to renal transplantation could be explained partly by the higher rate of immunization linked to pregnancies [31,32] that could limit the number of compatible organs and increase the risk of graft reject [29,33,34]. These disparities could also reflect the sex/gender effect on patient care. Indeed, women visit physicians more frequently than men [33]. Moreover, physicians’ practices differ in function of the patient’s gender [35,36,37,38,39,40]. For instance, studies in Italy and in the USA found that women are less likely to be informed about kidney transplantation options [41,42,43,44]. Gender/sex-based disparities have been observed also for access to transplantation, all organs considered [41,45,46]. Similarly, when living donation is considered, women are more likely to be living donors than men [32,33,42,47].

In 2015, the French Haute Autorité de Santé (HAS) published national recommendations for placement on the waiting list [48] to develop a shared strategy concerning the waitlisting process and to reduce disparities. In studies performed in Lorraine and Bretagne (two French regions), no gender-/sex-based disparity in the access to kidney transplantation was detected after adjustment for the patients’ characteristics [49]. However, in studies that included more French regions, the analyses adjusted for age and comorbidities showed that access to the waiting list and to kidney transplantation was lower for women [24,27,31]. This gender/sex difference was observed also in Ile-de-France, the French region with the highest waitlisting rate in France [23,50]. However, these geographical gender/sex disparities have not been studied extensively because they were not investigated in all regions, and at a smaller-scale level, such as the department-scale. Moreover, no study has compared access to kidney transplantation determinants in women and men, or investigated the effectiveness of the HAS national recommendations for the placement on the waiting list, nephrologists’ clinical practices, and potential gaps between recommendations and actual practices. Lastly, women’s and men’s perceptions and expectations about access to kidney transplantation have not been explored and compared. The aim of this work was to study women’s access to kidney transplantation in France and the reasons of potential sex/gender disparities.

## 2. Materials and Methods

### 2.1. Design

To better investigate the kidney transplantation access potential disparities between women and men in France, this research used a mixed methods approach that combines a quantitative study and a qualitative study [51]. The quantitative study describes and objectifies the factors associated with access to renal transplantation by analyzing data from the French national Renal Epidemiology and Information Network (REIN) [52,53] registry. The qualitative study will focus on the questions that cannot be assessed with a quantitative study (e.g., actors’ behaviors, action rationale, sense given to these actions) [51,54,55]. This complementarity will guide this study and its methodology.

**Figure 1:**
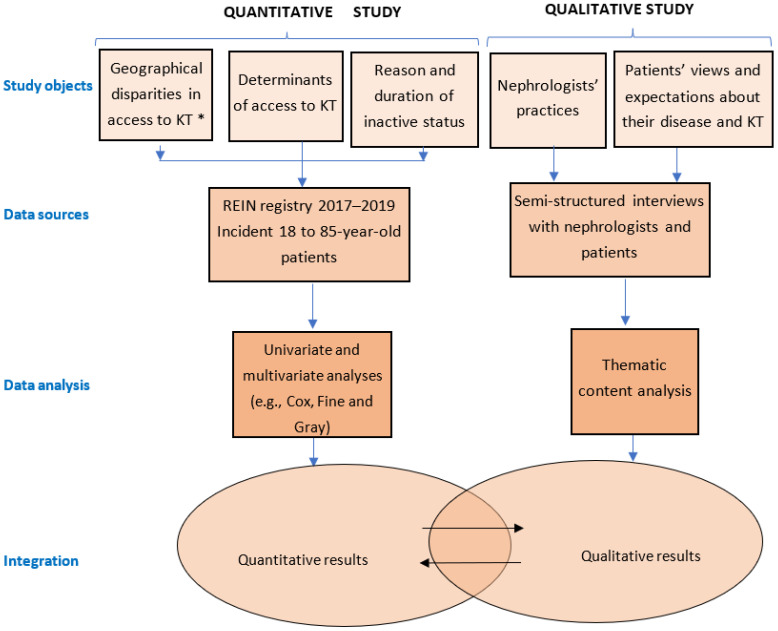
Mixed methods design. The quantitative and qualitative studies will be performed simultaneously. * KT: Kidney Transplantation (including access to the waiting list and access to kidney transplantation).

Several ways of mixing quantitative and qualitative methods have been described [51,54,56,57,58]. Here, qualitative and quantitative data were collected and analyzed simultaneously (Figure 1). Then, the results of both studies are integrated and discussed in view of the main objective, that is, to study women’s access to kidney transplantation in France. The methodologies of the quantitative and qualitative studies and the integration strategy are described in the next sections.

### 2.2. Quantitative Study

#### 2.2.1. Hypotheses

We hypothesized that access to kidney transplantation is not homogeneous in France. Specifically, access to kidney transplantation is not homogenous between women and men in some French territories (regions and departments) and the underlying reasons may differ among territories. We also hypothesized that the determinants of access to the waiting list and to transplantation as well as the reasons and duration of inactive status on the waiting list may be different between men and women.

#### 2.2.2. Objectives

The objectives of the quantitative study were: (i) to identify geographical disparities of access to kidney transplantation between men and women; (ii) to identify and compare the factors associated with placement on the waiting list and access to kidney transplantation between men and women; and (iii) to compare the reasons and duration of inactive status on the waiting list between men and women.

#### 2.2.3. Data

All 18 to 85-year-old patients with ESKD who started RRT in France in 2017, 2018 and 2019 were included and followed until 31 December 2021. Data will be extracted from the REIN registry. This registry was started in 2002 and includes all patients with ESKD treated by RRT in France [52,53,59]. Since 2012, all mainland regions and overseas territories are included. Data extracted from this registry by the Agence de la Biomedecine will include four categories of information: (i) the patient’s demographic data at RRT initiation: sex, age, occupational status, region, department, and census block of residence; (ii) the patient’s clinical and biological data at RRT initiation: primary kidney disease, physical disabilities, body mass index (BMI), walking difficulties, hemoglobin and albumin levels, comorbidities (e.g., diabetes, cardiovascular diseases, active malignancy, respiratory disease, and liver disease); (iii) RRT characteristics: date and modality of the first RRT, emergency or planned first dialysis, RRT facility ownership (public, private for-profit, or private not-for-profit), autonomous first dialysis session (home and out-of-center hemodialysis, non-assisted peritoneal dialysis; for patients who started dialysis in a training modality, the undergoing modality at the end of the third month will be considered); (iv) outcomes following RRT initiation: registration on the waiting list, transplantation, and death. Additional information will be included for waitlisted patients (date of waitlisting, blood group, anti-HLA immunization level, duration and reason of inactive status periods on the waiting list), for non-waitlisted patients (reason of non-inclusion: patient refusal, medical contraindication, or ongoing waitlisting process), and for kidney transplant recipients (date of procedure, donor type: living or deceased).

As this research focuses on the access to the first transplantation, patients who are considered for re-transplantation will not be included in the study.

Concerning the occupational status at RRT initiation, the REIN registry classifies patients as inactive (student, retired, at home) and active (unemployed, full-time and part-time employed). Consequently, this variable does not fully represent the patients’ socio-professional situation. As the REIN registry does not contain other data on the patients’ socio-economic status (e.g., education or income level), to overcome this limitation, the neighborhood socio-economic deprivation level at the census-block level (called IRIS in France) will be integrated using the European Deprivation Index (EDI) as a covariate [60].

#### 2.2.4. Analyses

First, the patients’ baseline characteristics will be described and compared between women and men using the Chi-squared test (qualitative variables) and *t*-test (quantitative variables).

Then, access to kidney transplantation (living or deceased donor) will be studied through the probability of occurrence of three events of interest: access to the kidney transplant waiting list, access to kidney transplantation after waitlisting, and access to kidney transplantation after dialysis starts. Associations between patients’ characteristics, including sex, and these three events of interest will be analyzed using Cox proportional hazard models [61] and also Fine and Gray competing risk models with death considered as the competing event for the three events of interest [62,63,64,65]. The Cox proportional hazard model will be used to study the etiological role of sex on the three events of interest, and the Fine and Gray model to predict women’s access to kidney transplants [66]. The Cox and Fine and Gay models assume that all patients may experience the event of interest. However, some patients with ESKD will never be registered on the waiting list or undergo transplantation. Moreover, these models do not determine whether women stay longer than men in dialysis before being included in the waiting list or undergoing transplantation. To overcome these limitations, “mixture models” [67,68] will also be used because they combine a logistic regression model to estimate the probability of waitlisting (or transplantation) and a conditional proportional hazard model to take into account the delay between dialysis start and waitlisting (or between waitlisting and transplantation, or between dialysis start and transplantation). Moreover, multilevel analyses will be performed to take into account random effects on the dialysis network’s level (i.e., dialysis centers that work together to offer all different RRT types in a given area) [69]. These analyses will be carried out successively at the national, regional, departmental, and census-block levels. Interactions between age, sex and neighborhood socio-economic deprivation (EDI) will be also examined. Patients registered on the waiting list before dialysis initiation will be considered as waitlisted at dialysis start. A *p*-value of <0.05 in multivariate analyses will be considered as significant.

Moreover, the factors associated with access to transplantation will be thoroughly investigated by integrating a spatial accessibility approach [70] using, as an indicator, the Index of Spatial Accessibility (ISA) [71] to nephrologists and transplantation centers at the census-block level. ISA, which is based on Enhanced Two-Step Floating Catchment Area (E2SFCA) approaches [72], provides a summary measure of two important and related components of accessibility: the volume of services available relative to the population’s size, and the proximity of the available services relative to the population’s location. For sensitivity analysis, ISA will be calculated with different decay functions (Downward Log Logistic, Gaussian, Exponential) that have been used in similar works [73,74]. Then, the associations of patients’ characteristics (including sex) and census-block level variables (EDI and ISA to nephrologists and transplantation centers) with the three outcomes of interest will be analyzed using a multilevel Cox shared frailty model. Interactions between age, sex, EDI and ISA will also be studied. All analyses will be performed using the Anaconda data science platform (Python and R distribution), Esri Network analyst, and STATA.

### 2.3. Qualitative Study

#### 2.3.1. Research Questions

For better understanding of the potential disparities in the access to kidney transplantation between women and men, we decided to trace back the patients’ care trajectory, and to determine the patients’ views and expectations about their disease and its management, particularly kidney transplantation. We will cross these data with the nephrologists’ practices regarding the HAS recommendations.

#### 2.3.2. Underlying Theory

The approach of our qualitative study is anchored in the interactionist movement [75] and specifically in the concept of “illness trajectory”, described by J.M. Corbin and A. Strauss [76]. Based on the study of chronic diseases, the concept of “illness trajectory” and the provided framework have the advantage to include “*not only the potential physiological development of an illness but also the work involved in its management, the impact of illness, and the changes in the lives of the ill and their families that in turn affect their management of the illness itself*” [77]. The process of managing a disease is anchored in a structural context that is the sum of the constraints that affect the actors’ choices and actions. Our position is to consider waitlisting as a step of a trajectory co-constructed and negotiated mainly between the patient with CKD and the nephrologist. The patients’ perception of their disease and of its management depends on this interaction and on the meanings that things have for them [75]. Therefore, it is relevant to study what surrounds the nephrologists’ practices, particularly the HAS recommendations, organ shortage and the effect of the local context of practice that can shape specific trajectory schemes. Moreover, it is pertinent to consider kidney transplantation using the concept of a “gift”, initially described by M. Mauss, and its three obligations: give, receive, give back [78]. In the case of kidney transplantation, patients “*must resolve the dilemma of receiving a gift–vital–without being able to thank the donor, give, or symbolically pay back*”, which can lead to some forms of culpability [79,80]. Patients must perform substantial psychological work to accept kidney transplantation. Our study will describe this work as well as the nephrologists’ role in it, and will determine whether this work is different between women and men.

#### 2.3.3. Study Population

The qualitative study will include patients with ESKD who started replacement therapy in 2021 and also nephrologists. Without seeking exhaustiveness, which is not feasible in a qualitative study, participants will be chosen using a purposive sampling methodology [81] based on the patients’ sex, age, and region of residence (Bretagne, Normandie, and Ile-de-France) to ensure heterogeneity. These three French regions have different profiles concerning disparities in the access to kidney transplantation. Unlike Ile-de-France, in Bretagne, despite a lower waitlisting rate, no sex-based disparity was found concerning the placement on the waiting list [23,49]. Additionally, ESKD epidemiology differs in these three regions. In 2017, ESKD incidence rates were 132 person per million (ppm), 156 ppm, and 194 ppm in Bretagne, Normandie, and Ile de France, respectively [82].

For the qualitative study, 40 patients (20 women and 20 men) and 15 nephrologists will be included in each region. Patients with contraindication to kidney transplantation according to the HAS recommendations (age > 85 years, active malignancy, BMI > 50 kg/m^2^, oxygen-dependent) will not be included [48]. Nephrologists will be chosen according to the facility ownership where they work, their years of experience, and their sex.

#### 2.3.4. Data Collected

Semi-structured interviews will be used to collect data because they allow a flexible interaction with the possibility to focus the discussion on the main themes but also to allow some freedom and initiative in the answers [83]. Data will be collected by clinical research assistants who have a good experience in qualitative studies and are not involved in patient care. They will be trained and will use previously tested interview guides (one for patients and one for nephrologists).

The patients’ interview guide (Supplementary File S1) starts by tracing back their “*illness trajectory*” [77]: How did the patient receive the diagnosis of CKD? Was the possibility of transplantation discussed before RRT? How does the patient manage the disease? Then, the patient’s consultations with a nephrologist, during which shared medical decisions can be made, also will be explored: What does the patient expect and retain from these consultations? How is the care decided? This will be followed by questions on the patient’s views about kidney transplantation and the work towards its acceptance. In addition, the patients’ socio-economic data (profession, education level) will be considered in these analyses.

The nephrologists’ interview guide (Supplementary File S2) will first investigate how these healthcare professionals see the standardization of medical practices (i.e., the 2015 HAS recommendations on waitlisting) and their level of knowledge and appropriation of these recommendations. Then, the nephrologists’ clinical practices will be explored to identify potential sex/gender differences: when and how is the subject of transplantation brought up with the patient? What are the choices made by the nephrologist and the underlying rationale?

All interviews will be audio-recorded and then transcribed. The approval by the local ethical committee and the interviewees’ consent will be obtained before data collection.

#### 2.3.5. Analyses

A thematic analysis will be carried out, as described by Burnard [84], using the Nvivo 12 software. An inductive approach with two coders will be implemented. The findings will be reported following the Consolidated criteria for reporting qualitative studies (COREQ) [85]. The first level of analysis will focus on the patients’ care trajectory, views and expectations about their care and access to kidney transplantation with a gender-based comparison. The analysis will focus particularly on kidney transplantation refusal by women and the reasons that can explain this decision. The second level of analysis will concern the nephrologists’ knowledge and appropriation of the HAS recommendations. Possible gaps between the HAS recommendations and the nephrologists’ clinical practice and their association with the patients’ access to kidney transplantation will be explored. This will allow us to determine whether nephrologists propose kidney transplantation less often to women than men. Moreover, each patient’s care trajectory will be traced back following the analytical framework proposed by Strauss and Corbin and its different phases [86]. *Trajectory schemes* will be identified in order to then analyze how the *trajectory workers*’ actions articulate. From this, a trajectory typology will be built as well as a nephrologist typology based on their practices and appropriation of the HAS recommendations. The two typologies will then be crossed to find links between practices and trajectories.

#### 2.3.6. Integration

The results of the quantitative and qualitative studies will be integrated by discussing them, looking for common concepts, and understanding how the qualitative approach can provide a deeper understanding of quantitative data. For example, the quantitative study may highlight high waitlisting rates in some regions, but significantly lower rates for women than men. Higher rates of patient’s refusal to waitlisting may also be found for women. Although these results are important, on their own, they do not give any insight into the underlying mechanisms and reasons. The qualitative analysis of nephrologists’ practices and the reconstitution of the patients’ care trajectories will enrich and explain these findings.

## 3. Discussion

This multidisciplinary study aims to understand more comprehensively the role of sex/gender as a health determinant and potential factor of disparity concerning access to healthcare, in this case, kidney transplantation. It involves specialists from different sectors (e.g., public health, epidemiology, nephrology, spatial analysis, sociology, and statistics) and combines two methodological approaches with different paradigms to improve knowledge on the access to kidney transplantation in France. To our knowledge, this is the first study using mixed methods to assess access to kidney transplantation in France with a special focus on the differences between men and women.

The mixed methods approach that combines quantitative and qualitative studies is becoming more frequent in scientific publications [87] and in nephrology studies [88]. This is a relevant approach for tackling some complex research questions, such as access to kidney transplantation. Specifically, the quantitative study will exploit the REIN registry data. However, despite its exhaustiveness, this registry does not provide information on patients’ healthcare consumption before RRT starts. On the other hand, the qualitative study will allow better understanding the patients’ pre-RRT care trajectory and how this could influence their access to kidney transplantation. Indeed, studies performed by our team in France showed that dialysis initiation in emergency is influenced by the care trajectory in the two years before dialysis start [89]. Therefore, it is important to study the patients’ care trajectory before RRT and how it may influence the patients’ current views and perspective about kidney transplantation. The qualitative study will allow us to reconstruct the care trajectory and follow-up through the patient’s point of view.

With a qualitative approach, researchers can study phenomena in their natural settings, and can try to make sense of or interpret them in terms of the meanings people give to them [90]. However, asking patients about their views and perspectives concerning kidney transplantation and their interactions with the nephrologist may quickly be perceived as a sensitive subject by some patients. This may lead to a “social desirability bias” [91], a term that describes “*the tendency to present oneself and one’s social context in a way that is perceived to be socially acceptable, but not wholly reflective of one’s reality*” [92]. Although this bias cannot be totally eliminated, several approaches can minimize it [93], including those used in this protocol: interview guides for data collection and interviewers not involved in the patients’ care.

This is the first mixed methods study on the potential association between sex/gender and access to kidney transplantation in France. The results will be presented at various working groups of the operational health agencies (HAS and Agence de la Biomédecine). This will help to produce robust evidence-based and relevant recommendations to enhance the access to kidney transplantation. The results will also be presented at meetings with regional health agency officials and healthcare professionals involved in kidney transplantation. This will contribute to develop more precise and efficient interventions, adapted to the local specificities of each region/department, to improve access to kidney transplantation, for example by encouraging waitlisting practices or reviewing policies on placement on temporary inactive status. Moreover, we will reach out to patients’ advocacy groups and will participate in patients meetings organized by these groups to inform patients on the results of our project, including the barriers to kidney transplantation access, and to promote discussion on this subject.

Finally, this type of project is important in the current societal context in which sex/gender inequalities persist in many areas and their reduction is a major social expectation and political issue.

## 4. Conclusions

This research protocol describes the use of a mixed methods approach to explore potential sex/gender disparities in kidney transplantation access.

## Supplementary Materials

The following supporting information can be downloaded at: https://www.mdpi.com/article/10.3390/ijerph192013524/s1, File S1: Patients’ interview guide, File S2: Nephrologists’ interview guide.
