# Supplementary material for: Women’s Access to Kidney Transplantation in France: A Mixed Methods Research Protocol
Source: Int J Environ Res Public Health. 2022 Oct 19;19(20):13524. doi: 10.3390/ijerph192013524 (PMC9603645; doi:10.3390/ijerph192013524)
Supplement: Supplementary file 1 [file ijerph-19-13524-s001.zip › ijerph-1953422-Supplementary File S1.pdf]

International Journal of
Environmental Research
and Public Health

Supplementary File S1: PATIENTS' INTERVIEW GUIDE

- To begin this interview, can you tell me about yourself and your activities?
- Can you describe how your disease started and its course?
  - ✓ The first signs
  - ✓ The diagnosis
  - ✓ Its impacts
- Disease perception: What do you think of your disease? Why do you think so?
- Tell me about your first visit to a nephrologist.
- Can you describe the follow-up after this first consultation?
  - ✓ Follow-up frequency
  - ✓ Difficulties encountered during the follow-up
- We will now talk about kidney transplantation. What do you know about kidney transplantation?
- Did your nephrologist talk with you about kidney transplantation?
- What do you know about living donor transplant?
- What do you know about the different steps required when planning a kidney transplant?
- Have you ever attended a group education session with other patients?
- How do you feel about kidney transplantation?
  - ✓ How do you consider transplantation?
  - ✓ Did you receive feedback on the advantages/disadvantages of transplantation?
  - ✓ How do think a kidney transplant may change your health?
- Did your doctor ever propose you to undergo transplantation?
- Are you in the process of having a pre-transplant work-up?
- How do you see the future?
