# Supplementary material for: Women’s Access to Kidney Transplantation in France: A Mixed Methods Research Protocol
Source: Int J Environ Res Public Health. 2022 Oct 19;19(20):13524. doi: 10.3390/ijerph192013524 (PMC9603645; doi:10.3390/ijerph192013524)
Supplement: Supplementary file 1 [file ijerph-19-13524-s001.zip › ijerph-1953422-Supplementary File S2.pdf]

International Journal of *Environmental Research and Public Health*

MDPI

Supplementary File S2: NEPHOROLOGISTS' INTERVIEW GUIDE

- To start this interview, could you tell me a little bit about your work as a nephrologist?
- Could you describe the first consultation with a patient with CKD (stage 4 and 5) whom you never saw before?
  - ✓ In which circumstances do they come to you ?
  - ✓ How are their feelings?
  - ✓ What kind of general information do you give to patients at the first consultation?
- In which context do you discuss the possibility of transplantation with your patients?
- Can you tell me about the information you give to patients about kidney transplantation and how they consider this information?
  - ✓ Do you tailor the information in function of the patient profile?
  - ✓ Do you give information on living donor transplantation?
- Do you suggest other information sources to your patients?
- Do you talk about transplantation with non-transplantable patients?
- Do patients ever refuse to undergo kidney transplantation?
  - ✓ What are the reasons for refusal?
  - ✓ How do you manage patients refusing kidney transplantation?
- How do you see the collaboration between you and the transplant team?
- We will now talk about the patient pre-transplant work-up. How do the different stages of this work-up take place?
- How do patients experience this process?
- The French National Authority for Health (HAS) often publishes recommendations on chronic kidney disease. What do you think of these recommendations?
- In 2015, HAS published good practice recommendations for kidney transplantation, including on access to the national waiting list. What do you think of these recommendations?
- What is your opinion about access to kidney transplantation for women in France?
- In your opinion, what should be done to improve the overall access to kidney transplantation in France? And particularly for women?
